# ‘Is it worth doing?’ Measuring the impact of patient and public involvement in research

**DOI:** 10.1186/s40900-015-0008-5

**Published:** 2015-07-31

**Authors:** Kristina Staley

**Affiliations:** TwoCan Associates, Wallace House, 45 Portland Road, Hove, BN3 5DQ UK

**Keywords:** Patient and public involvement, Service user involvement, Consumer involvement, Measuring impact, Evidence

## Abstract

**Abstract:**

Much of the current debate around the impact of patient/public involvement on research focuses on the lack of empirical data. While a number of systematic literature reviews have reported the various ways in which involvement makes a difference to research and the people involved, this evidence has been criticised as being weak and anecdotal. It is argued that robust evidence is still required. This review reflects on the use of quantitative approaches to evaluating impact. It concludes that the statistical evidence is weakened by not paying sufficient attention to the context in which involvement takes place and the way it is carried out. However, if scientific (systematic, quantitative, empirical) approaches are designed in a way to take these factors into account, they might not generate knowledge that is useful beyond the original context. Such approaches might not therefore enhance our understanding of when, why and how involvement makes a difference. In the context of individual research projects where researchers collaborate with patients/the public, researchers often acquire ‘new’ knowledge about life with a health condition. This new understanding can be described as experiential knowledge—‘knowledge in context’—that researchers gain through direct experience of working with patients/the public. On this basis, researchers’ accounts of their experience potentially provide a source of insight and learning to influence others, in the same way that the patient experience helps to shape research. These accounts could be improved by increasing the detail provided about context and mechanism. One of the most important contextual factors that influence the outcome of involvement is the researchers themselves and the skills, assumptions, values and priorities they start with. At the beginning of any research project, the researchers ‘don’t know what they don’t know’ until they involve patients/the public. This means that the impact of involvement *within any particular project* is somewhat unpredictable. The answer to the question ‘Is involvement worth doing?’ will always be ‘It depends’. Further exploration of the contextual and mechanistic factors which influence outcomes could give a stronger steer to researchers but may never accurately predict any specific impact.

**Plain English summary:**

In recent years, there has been considerable interest in finding out what difference patient and public involvement makes to research projects. The evidence published so far has been criticised for being weak and anecdotal. Some people argue we need robust evidence of impact from scientific studies of involvement. In this review, I consider examples of where impact has been measured using statistical methods. I conclude that the statistical evidence is weak, if the studies do not consider the context in which involvement takes place and the way that it is done. Studies designed to take this into account give us more confidence that the involvement did make a difference *to that particular project*. They do not tell us whether the same impact will occur in the same way in other projects and therefore have limited value. Researchers gain an understanding of involvement through their direct experience of working with patients and the public. This is ‘knowledge in context’ or ‘insight’ gained in the same way that patients gain expertise through their direct experience of a health condition. This means that detailed accounts of involvement from researchers already provide valuable learning to others, in the same way that patients’ insights help shape research. However, the impact of involvement will always be somewhat unpredictable, because at the start of any project researchers ‘don’t know what they don’t know’—they do not know precisely what problems they might anticipate, until the patients/public tell them.

## Introduction

In recent years, there has been much emphasis on developing the evidence base for patient and public involvement in research and a focus on demonstrating the impact of involvement. A number of systematic literature reviews have reported the various ways in which involvement makes a difference to research, the people involved and the researchers [1–4]. However, the current view is that much of this evidence is anecdotal, and therefore weak, and that more robust measures of impact are required [5, 6]. It is expected that evidence gained through empirical methods will provide a definitive answer to the question ‘Is public involvement in research worth doing?’ and identify if, when, where and how involvement brings benefits.

It is sometimes suggested that involvement is a right and therefore always of value irrespective of its impact. However, this does not mean that involvement cannot be usefully evaluated. We still evaluate processes that fulfil other rights, such as the democratic right to vote. In such cases, evaluation helps to understand ‘how to do it well’ as well as identifying meaningful outcomes for different stakeholders. In the context of involvement, I suggest that understanding involvement as a right only allows us to make the case that patients/the public should be ‘at the table’. I suggest understanding how involvement impacts on research quality adds another dimension to our understanding by defining who needs to be there and what role they should play. Therefore, I conclude that it is important to evaluate involvement to ask ‘What difference does it make?’ as well as ‘What’s the best way to do it?’

In this review article, I will argue that we are trying to address these questions in the wrong way. Working within health and social care research, our approach has been shaped by the values that underpin evidence-based medicine [7]. While this is valid and appropriate for testing healthcare interventions, this approach may not usefully apply to the evaluation (assessing the quality, merit or worth) of patient and public involvement in research, and the results may have little predictive value. This is because the impact of involvement is highly context dependent and the knowledge gained through involvement is experiential. One of the most important contextual factors that influence the outcome of involvement is the researchers themselves, in particular the skills, knowledge, values and assumptions they start with. They are often the ‘subjects’ who experience the impact of involvement. For this reason, the answer to the question ‘Is involvement worth doing?’ will always be ‘It depends’. We may increase our understanding of the range of factors that influence outcomes, by focusing our efforts on understanding ‘how it works’ rather than ‘what it achieves’. However, we might never be able to predict precisely what impacts involvement will have *within any particular research project*, because ‘we don’t know what we don’t know’ until patients/the public tell us.

## Review

### What counts as ‘evidence’?

Within the culture and ideology of evidence-based medicine, there is a hierarchy of evidence in which different research methods are ranked according to the validity of their findings [7]. Systematic reviews with meta-analyses of the results of high-quality randomised controlled trials (RCTs) are considered the gold standard, while individual case reports are ranked lowest. In the context of testing healthcare interventions, the use of RCTs is often valid and appropriate, because the target for the intervention is known, the intervention can be standardised and objective/quantitative data can be obtained about outcomes. The randomisation process within RCTs allows for contextual factors that may influence outcomes so that the outcomes can be attributed to one variable alone. The results therefore have predictive value in terms of the likely risks and benefits of a particular treatment. The evidence from RCTs is therefore often considered the best quality information to inform decisions about healthcare and health policy.

The healthcare researchers who are currently being encouraged to undertake patient and public involvement are steeped in this culture, and all stakeholders in the research process generally support the use of empirical research to underpin healthcare decisions. They all share a similar goal in wanting to improve research quality. It is therefore unsurprising that the debate around the impact of involvement is informed by these values, fuelling a quest to find similar kinds of evidence, i.e. to obtain quantitative data of impact obtained through systematic enquiry (see Table 1). However, I will argue that patient and public involvement is very different to the type of intervention usually assessed through RCTs, which may make such an approach less useful for the evaluation of its impact.

**Table 1:** The difference between ‘evidence’ and ‘experiential knowledge’

The nature of ‘evidence’	The nature of ‘experiential knowledge’
• Data obtained through systematic enquiry	• Knowledge ‘in context’ gained through experience—insight/wisdom
• Objective	• Subjective
• Rational	• Emotional
• Quantitative—measurement	• Qualitative—description

### The complex nature of patient and public involvement in research

Patient and public involvement in research is not a single activity—it takes many forms and operates at many different levels, strategic and operational and national and local. In the context of individual research projects, involvement can refer to activities as diverse as patients reviewing clinical trial protocols [8] through to social care service users presenting the results of research at a conference for practitioners [9]—as well as all that goes on at the different stages of research, in different types of research projects, involving people with very different kinds of experiences [1–4]. It includes consulting advisory groups with different kinds of membership, as well as involving single patients as co-researchers. This means it is very hard to standardise involvement. There is not a single, simple intervention we can test.

This also means that the impact of involvement is highly context dependent. If patients are involved in reviewing a clinical trial protocol then the impacts are most likely to be related to research design and recruitment strategies [8], but if they are involved in dissemination of research results, then the impact will most likely be on implementation and changes to practice [9]. We also know that the quality of the involvement process is key. If involvement is tokenistic, the chances of it making a difference are severely reduced [10]. For example, if involvement is restricted to asking the public to comment on a participant information sheet, they will not be able to influence any other aspect of the research process. Patients/the public may also make recommendations that researchers may decide to ignore. Therefore, the involvement process is less about the ‘method’ used to seek people’s views and more about what patients/the public are asked to contribute, what specific recommendations they make and what action is taken in response to their input.

In conclusion, given the complex nature of involvement, when we set out to evaluate its impact, we need to precisely define the form it takes, paying close attention to the context and the detailed mechanism [11], rather than using a loose definition ‘public involvement’ that in fact describes many different types of activity. A weakness of the current evidence of impact is that this detail is often missing, which limits our understanding of ‘why’, ‘when’ and ‘how’ the involvement has made a difference.

### Quantitative assessments of impact and their limitations

One of the commonly reported impacts of involvement is an increase in recruitment rates; for examples, see [9, 12–15]. This is an impact that is feasible to evaluate using quantitative methods, and two recent studies have reported using such an approach [16, 17]. Ennis and Wykes report that studies with greater involvement are more likely to reach at least 90 % of their recruitment target, while Johns reports ‘a small relationship between involving patients and reaching target recruitment’. Both studies were carried out within the National Institute of Health Research Clinical Research Network (NIHR CRN) and used similar approaches (see Table 2).

**Table 2:** Measuring the impact of patient and public involvement on recruitment to research

Example 1: Johns et al. [17] used data from the CRN’s Closed Study Evaluation Survey, which asks CRN researchers about involvement at different stages of their research projects and the perceived impact of the involvement from the researchers’ perspective. They also accessed CRN data relating to recruitment to time and target. Out of the 281 studies that responded to the survey, 193 had involvement of some kind, while 88 had no involvement. Two hundred and seventy-seven of the 281 studies had records about whether they had recruited to time and target. These data were analysed to explore whether involvement or other study factors (study design, trial randomisation, geographic scope, planned sample size or planned trial duration) had a significant relationship with recruitment. Involvement was the only factor that was shown to have a significant impact on the odds of achieving recruitment to time and target, although the effect was small.	
In the Evaluation Survey, one of the questions asks CRN researchers to indicate whether involvement improved the design/development of their study by selecting from a list of possible impacts. These include factors that have the potential to influence recruitment, such as improving engagement of seldom-heard groups and the quality of information provided to potential participants. Researchers can tick more than one option, and it is not clear where there is overlap. In 43 % of the studies in the sample (*n* = 83), researchers’ reported a specific change to recruitment procedures to make them more sensitive to participants’ needs.	
Example 2: Ennis and Wykes [16] analysed data from 374 studies registered on the Mental Health Research Network’s portfolio database. These data included information about researchers’ plans for involvement which are required as part of the application to join the portfolio. On the application form, MHRN researchers categorise the level of involvement in their project as being either consultation; researcher-initiated, jointly-initiated or patient-initiated collaboration; or patient-controlled research. The database also includes information about recruitment. Ennis and Wykes chose to define successful recruitment as reaching >90 % of the proposed recruitment target. A statistical analysis of these data showed an association between the description of involvement at the start of the study and successful recruitment, even after other factors such as study design, study complexity and clinical group under investigation had been taken into account. This effect was larger in studies with greater levels of involvement.	

Although both studies demonstrated a statistically significant association between involvement and recruitment, the effects are small and they did not identify causal links. This is because not enough attention was given to the context and mechanisms of involvement. Both studies compared one group of projects *with some kind of involvement* with another group of projects with no involvement. They controlled for other contextual factors that might influence recruitment, such as planned sample size, but not for the variation within the involvement itself. In the case of Ennis and Wykes, we do not know exactly what type of involvement took place, what specific recommendations the patients/public made and whether these were related to recruitment and which of these suggestions the researchers took on board. In the case of Johns et al., again, we do not know the details of how the involvement took place. We do know that in 43 % of the projects within the sample, reported impacts specifically related to recruitment processes, which could mean there was no impact on recruitment in the remaining 57 %. Perhaps most importantly, in both these quantitative studies, we do not know if the projects within the sample anticipated or experienced any barriers to recruitment. Patient and public involvement would not have impacted on recruitment where there was no pre-existing problem.

Both studies have clearly made best use of the data currently available. However, they may have weakened the results by using a loose definition of patient and public involvement that combined the results from projects that may have used different involvement approaches, in different contexts, leading to very different kinds of impacts. The authors of both studies concluded that more research is needed to understand ‘the variation in levels and types of PPI in different types and design of study’ (i.e. the context) [17] and the ‘specific *mechanism* by which patient involvement seemingly improves recruitment’ [16] (emphasis added). It could be argued that an RCT designed to test the impact of patient and public involvement on recruitment within a specified context (i.e. where there is an anticipated problem with recruitment) and a specified mechanism (i.e. a change to recruitment processes identified via patient and public involvement) might give much stronger statistical evidence of impact and a clearer indication of cause and effect.

### The potential of RCTs to evaluate the impact of involvement

Based on the arguments presented above, in this section, I explore the potential use of an RCT designed to assess whether improvements to recruitment can be attributed to interventions put in place in response to involvement, drawing on a case study of service user involvement in social care research [9, 18].

In this case study, the research project aimed to improve support services for families affected by compulsory adoption. This included the birth families whose children had been taken into care and adopted and the families who had adopted those children. Working with birth families presented a number of challenges including recruitment, and the researchers were aware they needed to approach this project with a great deal of sensitivity. They therefore set up an advisory group of birth relatives who were involved throughout the project. The group was asked to comment on the recruitment strategy and they made a number of suggestions. They altered the leaflet sent to potential participants and commented on the practical approach. The researchers planned to recruit some of the service user participants via social services, who were sending out letters on their behalf. The advisory group recommended avoiding use of the social service franking machine, as any letter they received with this postmark would be thrown away without being opened. They suggested using a stamp instead. The researchers made these changes. Although they could not prove these changes directly influenced recruitment, they reported that theirs was the only project amongst their peers that had no problems with recruiting [18].

It would be simple to design an RCT that could test out the relationship between the service users’ recommendations and recruitment. For example, the researchers could have chosen to work with say six different local authorities, used the franking machine in three cases (selected at random) and used a stamp for postage at the three others. A similar approach could have been used to assess the impact of the service users’ input into the project leaflet, comparing the original with the new version. Then recruitment rates across the six organisations could have been analysed quantitatively. Conceivably, this might generate highly significant statistical evidence of a link between the changes made as result of involvement and the recruitment rates at the different organisations. It might even have told us that making both sets of changes (using a stamp and changing the leaflet) produced the most significant change.

If such evidence was obtained, what new learning would it provide? It would give us confidence that the involvement did indeed make a difference to recruitment, but only *in the context of this particular project*. We could not conclude that such an approach would make a significant difference to all service users or in the context of say NHS organisations contacting patients. Nor does it tell us that these are the mechanisms by which involvement always influences recruitment. Therefore, I suggest that the evidence would have little predictive or explanatory value, beyond what we have already learnt from the researchers’ report.

Would this evidence make a difference to the researchers in this case study? They already had sufficient confidence in the service users’ expertise to adopt their suggestions in full. Furthermore, they might not have wanted to risk slowing recruitment by not implementing these changes. So why would they want to invest time and resources in such an experiment?

On a more general note, would the large-scale investment in scientific approaches to test out all the various ways in which involvement has an impact on research be worthwhile? I suggest not, because the findings would not reveal when or how involvement makes a difference. We would still be struggling to answer a researcher who says, ‘Is it worth involving patients/the public in my project?’

### The impact of involvement as experiential knowledge

Perhaps one of the most important contextual factors that influence the impact of a collaborative form of involvement in research projects is the researchers themselves and crucially what skills, knowledge, values and assumptions they start with. For example, if a researcher is good at writing in plain English, then involvement in review of patient information may not make much of a difference to whether it is easy to understand [19]. If the researcher has made assumptions about what treatment outcomes are important to patients, then involvement in trial design may bring about very significant changes in outcome measures [20–24]. If the researcher develops a research strategy that somehow dissuades potential participants, involvement may make a significant difference to recruitment [9].

In practice, it is therefore often the researcher who directly experiences the impact of involvement. It is their thinking, planning, values and communication that are often challenged through involvement, a process that researchers describe as ‘a lightbulb moment’ or ‘reality check’ [21]. For this reason, the impact of involvement could be more usefully conceived as a form of experiential knowledge. In the same way that patients’ knowledge is gained through direct experience of a health condition, researchers’ understanding of involvement is gained through their direct experience of working with patients/the public. Knowledge about how involvement makes an impact is therefore ‘knowledge in context’, which is different in nature and quality to ‘evidence’ obtained through systematic enquiry (Table 1).

This subjective nature of the impact of involvement is one of the main reasons that its impact is somewhat unpredictable *for any given project*. Researchers will not be aware of any problems, misassumptions, practical difficulties with participating in their proposed study, etc. until they have actually involved the patients/the public. For example, how could they know if their recruitment strategy is problematic for the people they are trying to recruit without first talking to those same people to identify the barriers? At the start of any research project, the researchers ‘don’t know what they don’t know’, and therefore, we cannot predict which particular inputs from patients/the public are going to be most beneficial.

### What do we still need to learn about the impact of patient and public involvement?

All the recent reviews of the literature on the impact of patient and public involvement have consistently identified the same ways that involvement makes a difference to research projects. For example, in 2009, a review of 89 articles reporting on impact identified nine categories of impact (Table 3) [4]. Since then, the involvement literature has been reviewed and summarised for the INVOLVE evidence library [25]. During the intervening 6 years, an additional 118 articles and reports have been included in the database, all of which have reported the same kinds of benefits and downsides to involvement. No new categories of impact have emerged. This suggests that we may have reached saturation in terms of describing the impacts on research projects, and we may gain more from focusing our attention elsewhere.

**Table 3:** Reported impacts of involvement on research

Categories of impact identified through literature reviews
1. Impact on the research agenda—the topic, research question and funding decisions
2. Impact on research design and delivery—influencing the research design, tools and choice of method, recruitment, data collection and analysis, writing-up and dissemination.
3. Impact on research ethics—the consent process and developing ethically acceptable research
4. Impact on the people involved
5. Impact on the researchers
6. Impact on participants
7. Impact on the wider community
8. Impact on community organisations
9. Impact on implementation and change

The gap in understanding that remains is how these impacts/outcomes are achieved in different ways in different contexts and when they most frequently occur. For example, patient and public involvement has been reported to boost recruitment through many different means, including accessing seldom-heard communities, making invitation letters and participant information sheets easier to understand and making participation more attractive through improving the practical arrangements for participants [9, 12–15, 26]. Further exploration of when these different types of intervention are put in place could improve our capacity to identify the contexts in which they are likely to be of value. This could enable to give us a stronger steer to researchers on when this form of involvement is likely to be of benefit.

## Conclusions

The current perception of the published evidence relating to the impact of patient and public involvement in individual research projects is that it is weak and needs to be strengthened through use of quantitative or scientific methods. The expectation is that this will provide more robust evidence and will help us to be clearer about when involvement is most effective. Such arguments may be being driven by the values underpinning evidence-based medicine. In this review, I have argued that while such methods are entirely appropriate and valid for assessing healthcare interventions, they do not usefully apply to the evaluation of a highly context-dependent and complex process such as involvement in research. Quantitative and experimental approaches that do not incorporate contextual factors and variation in involvement processes into their design may weaken the evidence of impact or produce no evidence of impact [19]. When such scientific approaches are designed to test the impact of specific involvement interventions within specific contexts, the results may be more robust, but the lesson is of little predictive value *beyond the original context*. Therefore, our understanding of when involvement works best may not be much enhanced by quantitative approaches.

The impact of involvement in individual research projects where researchers collaborate with patients/the public might be more usefully conceived as a form of experiential knowledge, expertise that is gained through the researchers’ direct experience of working with patients/the public. With this understanding, researchers’ accounts of involvement provide a source of insight and learning that might usefully inform the approaches used by others, in the same way that insights and learning from the patient experience can usefully shape research processes. For example, the lessons from the case report described above suggest that involvement in recruitment strategies is likely to be beneficial in the context of recruiting service users who feel stigmatised by, excluded from or even hostile to services/service providers.

However, researchers’ accounts of involvement to date have not been sufficiently detailed, in that they do not always describe the context, mechanism and expected outcome of any chosen approach. These details are important to understanding the potential causal links [11]. For example, many reports in the literature describe the research context (aim and type of study), the means by which patients’/the public’s views and opinions were obtained (through setting up an advisory group, membership of a study management team, a single patient co-applicant, etc.) and a broad description of impact (improved the survey questionnaire, increased recruitment, etc.). To understand ‘how it works’, I suggest we need more details about the following:

- where the researchers started—their original plans, priorities, values, assumptions
- what recommendations were made by the public/patients and why
- what changes were made in response—which recommendations did the researchers take on board and why
- what outcomes were observed
- What outcomes were observed by researchers and patients/the public

More detailed accounts of this kind would provide a rich source of learning that could explain how involvement works in different contexts. We already have a clear and consistent picture of the downstream impacts of involvement on research, for example, better recruitment rates and clearer participant information sheets. We now need to understand the different ways these outcomes are achieved.

A more fundamental issue is that the outcome of any particular involvement activity may always be somewhat unpredictable, because researchers cannot anticipate the problems in their approach, because of their lack of knowledge of life with a health condition. This is the reason why they need input from patients/the public. The answer to the question ‘Is involvement worth doing?’ will always be ‘It depends’. However, with the wisdom and insight we gain from researchers’ detailed accounts and accumulated experience, we may be able to give a much clearer explanation of the factors that a successful outcome depends on.
